# Functional interactors of three genome-wide association study genes are differentially expressed in severe chronic obstructive pulmonary disease lung tissue

**DOI:** 10.1038/srep44232

**Published:** 2017-03-13

**Authors:** Jarrett D. Morrow, Xiaobo Zhou, Taotao Lao, Zhiqiang Jiang, Dawn L. DeMeo, Michael H. Cho, Weiliang Qiu, Suzanne Cloonan, Victor Pinto-Plata, Bartholome Celli, Nathaniel Marchetti, Gerard J. Criner, Raphael Bueno, George R. Washko, Kimberly Glass, John Quackenbush, Augustine M. K. Choi, Edwin K. Silverman, Craig P. Hersh

**Affiliations:** 1 Channing Division of Network Medicine, Brigham and Women’s Hospital, Boston, MA, USA; 2 Division of Pulmonary and Critical Care Medicine, Brigham and Women’s Hospital, Boston, MA, USA; 3 Department of Medicine, New York Presbyterian/Weill Cornell Medical Center, New York, NY, USA; 4 Department of Critical Care Medicine and Pulmonary Disease, Baystate Medical Center, Springfield, MA, USA; 5 Division of Pulmonary and Critical Care Medicine, Temple University, Philadelphia, PA, USA; 6 Division of Thoracic Surgery, Brigham and Women’s Hospital, Boston, MA, USA; 7 Department of Biostatistics and Computational Biology, Dana-Farber Cancer Institute, Boston, MA, USA.

## Abstract

In comparison to genome-wide association studies (GWAS), there has been poor replication of gene expression studies in chronic obstructive pulmonary disease (COPD). We performed microarray gene expression profiling on a large sample of resected lung tissues from subjects with severe COPD. Comparing 111 COPD cases and 40 control smokers, 204 genes were differentially expressed; none were at significant GWAS loci. The top differentially expressed gene was *HMGB1*, which interacts with *AGER*, a known COPD GWAS gene. Differentially expressed genes showed enrichment for putative interactors of the first three identified COPD GWAS genes *IREB2, HHIP*, and *FAM13A*, based on gene sets derived from protein and RNA binding studies, RNA-interference, a murine smoking model, and expression quantitative trait locus analyses. The gene module most highly associated for COPD in Weighted Gene Co-Expression Network Analysis (WGCNA) was enriched for B cell pathways, and shared seventeen genes with a mouse smoking model and twenty genes with previous emphysema studies. As in other common diseases, genes at COPD GWAS loci were not differentially expressed; however, using a combination of network methods, experimental studies and careful phenotype definition, we found differential expression of putative interactors of these genes, and we replicated previous human and mouse microarray results.

Chronic obstructive pulmonary disease (COPD) is characterized by progressive airflow obstruction accompanied by chronic inflammation. It is the third leading cause of morbidity and mortality worldwide^1^. Although cigarette smoking is the major risk factor, multiple studies have demonstrated a genetic component to COPD susceptibility^2^,^3^,^4^,^5^. Genome-wide association studies (GWAS) have identified multiple genetic loci associated with COPD susceptibility, with replication of the results across different populations^6^,^7^,^8^.

Many published studies have performed microarray gene expression profiling in COPD lungs^9^,^10^,^11^,^12^,^13^,^14^,^15^,^16^,^17^. In contrast to the GWAS, there has been minimal overlap between the differentially expressed genes identified in each microarray study^18^; greater overlap has been found for differentially expressed pathways. These microarray studies have been limited by variable definitions of COPD, incomplete consideration of past and current smoking status, failure to consider quantitative traits and COPD heterogeneity (such as emphysema and airway disease), and until recently, small sample sizes^19^,^20^. The COPD microarray studies have not found robust differential expression of the genes at GWAS loci, which has been shown in other complex diseases and traits, including coronary heart disease, height, type 2 diabetes, and autism^21^,^22^,^23^,^24^. Although most GWAS single nucleotide polymorphisms (SNPs) are likely regulatory variants and perhaps eQTLs^25^,^26^,^27^,^28^, the magnitude of effect of these SNPs on gene expression may be too subtle to capture with genome-wide expression studies in disease tissue.

We hypothesized that the genes identified at COPD GWAS loci (GWAS genes) may point to pathways and interacting gene networks important for COPD pathogenesis, and that genes in these pathways may be differentially expressed in lung tissues. However, the pathways relevant for many COPD GWAS genes are largely unknown. Therefore, we focused on the first three identified COPD GWAS genes, which have the strongest association signals: *IREB2* (iron responsive element binding protein 2), *HHIP* (hedgehog interacting protein) and *FAM13A* (family with sequence similarity 13 member A)^6^, for which multiple omics datasets have been previously generated. Other COPD GWAS genes lacked the breadth of omics data required for a similarly thorough study. We used these available data, from *in vitro* and *in vivo* models^29^,^30^,^31^,^32^ and *in silico* analyses, to identify gene sets functionally related to these genes. We merged these results with gene expression profiling from human COPD lung tissues and former smoker controls to demonstrate that the genes related to the GWAS genes, but not the GWAS genes themselves, were differentially expressed in the disease-relevant tissue.

A Weighted Gene Co-expression Network Analysis (WGCNA) was performed to identify gene modules associated with COPD status, as network medicine approaches^33^,^34^ provide insight into the understanding of complex disease and have been applied to the study of COPD^32^,^35^,^36^, heart disease^21^, diabetes^23^ and autism spectrum disorder^24^. By leveraging these methods in lung tissue, and examining interacting gene set enrichment, co-expression networks and pathways instead of individual genetic determinants, this study sought to identify key genes and gene modules involved in the molecular pathogenesis and etiology of COPD.

## Results

### Gene expression association with phenotype variables

We performed microarray gene expression profiling on lung tissue samples from 111 COPD cases and 40 control smokers with normal lung function (Supplemental Table S1). The differential expression analysis using R and the package limma (see Methods) identified 214 probes (mapped to 204 genes) associated with COPD at a 5% FDR; the top results are provided in Table 1 and all significant results may be found in Supplemental Table S2. The top differentially expressed probe is annotated to *HMGB1*. This probe was also the top gene expression association with lung function measures forced expiratory volume in 1 sec (FEV_1_)% predicted and the ratio of FEV_1_ to forced vital capacity (FVC). None of the previously identified genome-wide significant COPD GWAS genes (p-value < 5e-8) from the NHGRI-EBI Catalog (www.ebi.ac.uk/gwas/) were significantly differentially expressed (Table 2). A summary of the results for the other COPD-related phenotypes is provided in Supplemental Table S3. There were 1,556 differentially expressed probes (mapped to 1,366 genes) at a 5% FDR for FEV_1_%predicted and 1,689 differentially expressed probes (mapped to 1,429 genes) for the ratio of FEV_1_ to FVC. In lung function analyses in COPD cases only, the numbers of differentially expressed probes were lower and the overlap with the results for the full cohort was modest (Supplemental Figure S1). However, nearly half of the case-only results are recapitulated in the full-cohort analyses.

Pathway enrichment tests were performed using the gene symbol annotations for the 214 probes (204 unique genes) differentially expressed by COPD case/control status (Supplemental Table S4). The two nominally significant Reactome results (FDR ≤ 0.1) were *Regulation of actin dynamics for phagocytic cup formation::2029482* and *Fcgamma receptor (FCGR) dependent phagocytosis::2029480*. The higher false discovery rate threshold was chosen to highlight pathways or processes that may be of interest in future studies. The GO results identified a broad group of pathways, with processes related to protein modification and apoptosis being dominant.

### Interactor enrichment in differentially expressed genes

While we did not observe differential expression of genes at GWAS loci, we noted that the top differentially expressed gene, *HMGB1* (high mobility group box 1), has been identified as an important interacting partner of *AGER* (advanced glycosylation end product specific receptor)^37^, a gene implicated by GWAS for emphysema susceptibility^38^,^39^,^40^. This led us to test whether interacting partners of other COPD GWAS genes were also differentially expressed. The available *in vitro, in vivo*, and *in silico* datasets, described in the Methods section, defined sets of genes that may interact with COPD GWAS genes *HHIP, FAM13A*, and *IREB2*. These were tested for enrichment within the 204 COPD differentially-expressed genes. Table 3 shows that the genes differentially expressed in COPD lungs are enriched for interacting partners of *IREB2, HHIP* and *FAM13A*. There was similar enrichment in the 1,366 genes with expression associated with FEV_1_% predicted and the 1,429 genes associated with FEV_1_/FVC ratio. In the UpSet^41^ plot of the intersecting genes across the enrichment results (Supplemental Figure S2), the rows correspond to the rows of Table 3, and the gene sets for each row are the genes that overlap with the 204 differentially expressed genes (column three of Table 3). The intersecting genes for each column are listed in Supplemental Table S5. *HMGB1* and *CD79A* were present in the intersection of *IREB2* RNA immunoprecipitation sequencing (RIP-seq) and the *FAM13A* trans-eQTL overlaps, and *SERPINE2* was found in the overlap for *IREB2* RIP-seq (Supplemental Table S5 and Supplemental Figure S2). *POU2AF1* and *BCL11A* were present in overlaps involving two different *Hhip*^+/−^ mouse smoking models. *BCL2* was found in the intersection of gene list overlaps for an *Hhip*^+/−^ mouse model, *IREB2* RIP-seq and *FAM13A* trans-eQTL.

In a COPD GWAS dataset^8^, nominal association with case-control status was observed for SNPs annotated to *BCL2* (rs1481031, p = 0.0003), *BCL11A* (rs191541310, p = 0.007), *POU2AF1* (rs2282637, p = 0.008), *HMGB1* (rs117938771, p = 0.01), and *SERPINE2* (rs6721140, p = 0.03). No association was observed for *CD79A* (rs112580282, p = 0.2).

### Cis-eQTL analysis

For the *HHIP* (rs11724319, p = 0.03) and *FAM13A* (rs2609255, p = 0.001) eQTLs, the risk allele dosage corresponds to increased expression, while for the *IREB2* eQTL (rs2009746, p < 1.0e-10) the risk allele dosage corresponds to decreased expression^8^.

### Network analysis

**Figure 1:**
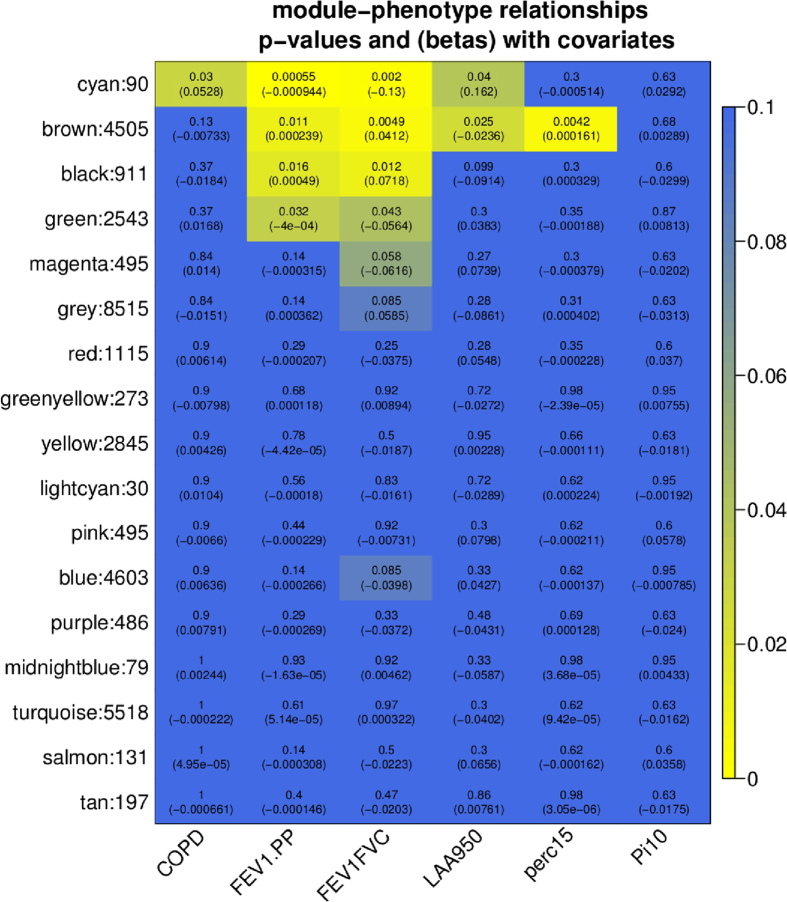
Heatmap of module association with phenotype variables (color scale for adjusted p-value). The top number in each cell corresponds to the FDR q-value and the bottom number is the beta coefficient from the linear regression model. COPD = COPD case-control status, FEV1.PP = forced expiratory volume in 1 sec, percent predicted; FEV1FVC = ratio of FEV1 to forced vital capacity (FVC); LAA950 = Low attenuation areas at −950 HU on chest computed tomography (CT) scans; perc15 = 15th percentile of the lung density histogram on chest CT scans; SRWA-Pi10 = square root wall area of a hypothetical airway with 10mm internal perimeter. The phenotype variables FEV1, FEV1FVC and perc15 decrease with COPD, while LAA950 and SRWA-Pi10 increase with disease.

**Figure 2:**
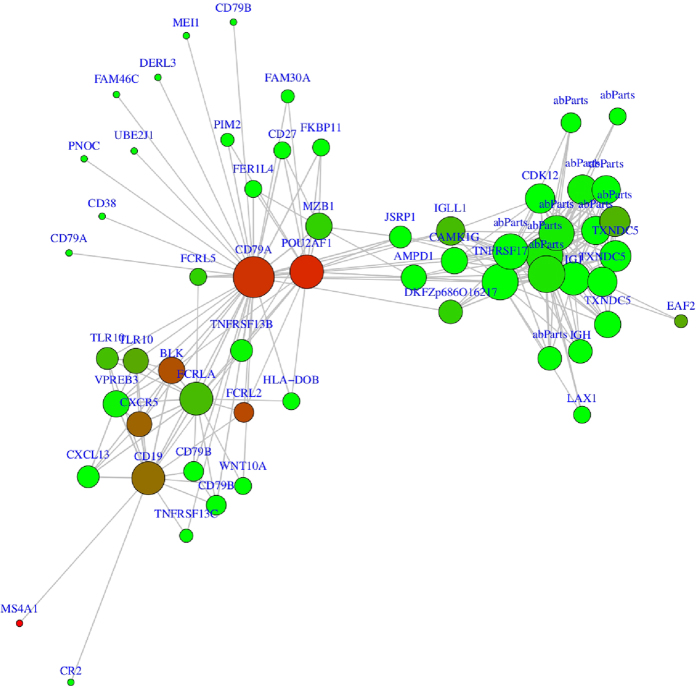
Module that was significantly associated with COPD case-control status (cyan module sub-network). Node size is proportional to the node degree and the color is related to the p-value in the differential expression analysis for COPD status (red lower p-value and green higher p-value).

A weighted gene co-expression network was constructed to group together co-expressed genes prior to pathway analysis^42^,^43^. The final network consisted of 17 modules, ranging in size from 30 to 5,518 probes (Supplemental Table S6). There was high correlation within the modules as expected, with the mean module membership (MM), or mean correlation of gene expression with the module eigengene, varying from 0.53 to 0.79 across all modules. The grey module is a grouping of probes with outlying gene expression profiles and was not considered further. Tests of association between phenotype variables of interest and the module eigengenes were performed for each model (see Methods), and the results were summarized in a heatmap (Fig. 1). Only the cyan module (number of probes = 90) was significantly associated with COPD case-control status (FDR < 0.05); however, the cyan module and brown module (number of probes = 4505) were associated with multiple COPD-related phenotypes, including case-control status, two measures of lung function – FEV_1_% predicted and FEV_1_/FVC ratio – and emphysema, measured as low attenuation areas <−950 HU on chest computed tomography (CT) scans. The probes within the cyan module and the top probes from the brown module after sorting by gene significance and module membership values, are listed in Supplemental Tables S7 and S8; putative driver genes for the two modules are highlighted at the top. We define driver genes by gene significance (GS) FDR <0.05 in COPD differential expression analysis and higher module membership (MM) correlations. These include *CD79A* (GS = 0.03, MM = 0.93) and *POU2AF1* (GS = 0.03, MM = 0.91) for cyan, and *HMGB1* (GS = 1.77e-5, MM = 0.84) for the brown module. The probes annotated to *AGER* were found in the grey module, and therefore co-expression with other genes, including *HMGB1*, was not observed (Pearson r = 0.09 for *HMGB1:AGER*). The gene network in the cyan module is shown in Fig. 2. In this module, *CD79A* and *POU2AF1* have high degree (high number of edges) and therefore appear as hubs. Seventeen genes in this module overlap with the mouse *Hhip*^*+/+*^ vs. *Hhip*^+/−^ 6 month smoking model gene set (Supplemental Table S7, enrichment p-value 6.5e-14), demonstrating that pathways associated with lung damage and B cell aggregate formation^32^ are shared across species. This module includes 9 genes (15 probes) associated with emphysema in a previous lung tissue microarray expression study^10^ and 14 genes (17 probes) found to be differentially expressed between emphysema and bronchiolitis^44^. The brown module was relatively large and did not demonstrate sub-structures like those observed in the cyan module; sub-structures could have informed a network pruning step. In the COPD case-control analysis, the top probes annotated to *HHIP, FAM13A* and *IREB2* were found in the grey, grey and blue modules, respectively.

Pathway enrichment tests were performed for the genes in the cyan and brown modules (Supplemental Tables S9 and S10). In contrast to the pathway enrichment results for the top COPD associated genes, the pathways enriched in the cyan module are more specific, demonstrating enrichments for B cell related processes. For the brown module, the results are mixed and broad, highlighted by RNA processing, apoptosis, immune system, and protein modification pathways.

## Discussion

In a microarray study of surgically-resected human lung tissue from ex-smoking severe COPD subjects and controls, we discovered 214 differentially expressed probes, corresponding to 204 unique genes. We found that none of the significant genes from previous COPD GWAS in the NHGRI-EBI Catalog or emphysema GWAS^39^ were differentially expressed. As the top differentially expressed gene was *HMGB1*, a known interactor of the COPD and emphysema gene *AGER*, we sought to determine whether there was differential expression of the putative interactors for the first three identified COPD GWAS genes: *IREB2, HHIP* and *FAM13A*. Using a combination of experimental datasets and network analyses, we observed strong enrichment for putative interactors of *IREB2, HHIP*, and *FAM13A*. We identified a gene co-expression module strongly associated with COPD, and enriched for B cell functions. This module contained seventeen overlapping genes compared to the *Hhip*^+/−^ smoking mouse model^32^, highlighting a cross-species signal of B cell related pathogenesis in COPD and emphysema. Using the strict phenotype definition of severe COPD in former smokers, we were able to replicate significant gene and B cell pathway findings from a previous emphysema gene expression study^10^, in contrast to the poor replication in many previous COPD microarray studies^18^.

Our top differentially expressed gene was *HMGB1*. The protein encoded by *HMGB1* is an interacting partner of *AGER* (which encodes the emphysema biomarker sRAGE), a replicated lung function and emphysema associated gene^38^,^39^,^45^. Plasma HMGB1, along with sRAGE, has been found to be higher during acute exacerbations in COPD patients and lower during convalescence^46^. Increased HMGB1 has been observed in blood and lung of smokers with COPD relative to healthy smokers^47^. There is also evidence of elevated HMGB1 protein levels in the sputum^48^ and bronchoalveolar lavage (BAL)^37^ of COPD patients. The nature of *HMGB1* involvement in COPD is unclear^49^, and the reduced expression of the *HMGB1* gene in our COPD lung tissue study, in contrast to the increased protein levels in sputum and BAL in these previous studies, and in a study of plasma levels in COPD patients^50^, requires further investigation; differences in smoking status, disease severity and tissue of origin may explain the differing results. *HMGB1* was a driver gene in the brown module, which was associated with COPD, lung function and emphysema.

Our analysis also identified interacting genes that may play a role in COPD pathogenesis. *SERPINE2* (sepin peptidase inhibitor clade E member 2) has been suggested as a COPD and asthma susceptibility gene^51^,^52^, and *CD79A* (CD79a molecule, immunoglobulin-associated alpha) was identified as a putative driver gene for the cyan module. Another putative driver for the cyan module, *POU2AF1* (POU class 2 associating factor 1), was present in the intersection with the *Hhip*^+/−^ mouse smoking model. *BCL2* (Bcell CLL/lymphoma 2) has been implicated in COPD via regulation of apoptosis through mitochondrial maintenance functions^53^,^54^,^55^.

Construction of co-expression networks is a method to group similarly expressed probes into correlated network modules, with potentially common functions. Using WGCNA, we identified modules associated with COPD, lung function and quantitative CT emphysema. The cyan module was most significantly associated with COPD status, and the pathway enrichment analyses provided insight into the functions of the module genes, namely B cell activation, proliferation, aggregation and signaling. This is consistent with the histologic findings of lymphoid aggregates in severe COPD patients and the importance of immune pathways in COPD^56^,^57^,^58^,^59^,^60^,^61^. In addition, lymphoid aggregates were observed in the lungs of the *Hhip*^+/−^ smoking mouse^32^; the overlap of 17 genes in the cyan module with the mouse *Hhip*^+/+^ vs. *Hhip*^+/−^ 6 month smoke gene set provides further evidence of a cross-species signature of B cell involvement in COPD.

In a previous study restricted to former smokers, B cell receptor signaling pathways were enriched in genes whose expression was associated with emphysema severity^10^. Nine of the 127 genes that were differentially expressed in the Campbell *et al*. study are found in our cyan module, demonstrating replication of the B cell signature. A key member of this overlap set is *CD79A*, which is a hub gene in the cyan module. The gene *BCL11A* (B-cell CLL/lymphoma 11A) was also replicated. *BCL11A* is a transcription factor critical to B cell development^62^,^63^. In contrast to previous COPD gene expression studies^20^, our study and the Campbell *et al*. study both included subjects with severe COPD and eliminated the effects of current smoking on gene expression by limiting to former smokers^64^,^65^, allowing for replication of results at both the gene and pathway level. The second putative driver gene for the cyan module *POU2AF1*, was also identified in a study of emphysema by Faner *et al*.^44^ and is a B-lymphocyte specific transcription factor^66^. In this recent study, also of former smokers^44^, we observed replication of our results at the gene level.

In COPD and other complex diseases, there is often little overlap between genes at GWAS loci and differentially expressed genes in the target tissue^20^,^21^,^22^,^23^,^24^, despite the fact that most GWAS associations are likely due to regulatory variants. We found that genes that potentially interact with COPD GWAS genes were differentially expressed. These GWAS genes identified relevant pathways, but they themselves were not differentially expressed. It has been shown that GWAS genes tend to be located on the periphery of gene interaction networks and are not hub genes^67^; GWAS genes were not found in the hubs of our co-expression modules. Genetic variants affecting hub genes may be too deleterious to cells to be maintained in the population. Using network and pathway methods, we are able to capture the overall genetic perturbation and relate it to the gene expression analysis.

We demonstrated significant differential gene expression related to COPD in this study; however increased sample sizes in future studies will improve the power for gene identification. Inclusion of subjects with mild to moderate COPD will be necessary to determine whether B cells and other pathways are relevant earlier in the disease course, as infections are a common component of more severe COPD. Additional CT scan data in future studies should allow us to dissect COPD phenotypes of emphysema and airway disease. Data from specific cell types would provide more specificity than homogenized lung tissue. In addition, the variation in significance seen across our interactor enrichment results was not surprising given the different potential mechanisms of interaction and the heterogeneity with respect to species and cell types. Future studies may involve other COPD GWAS genes beyond the top three in the current study, as additional omics datasets are generated for those genes. Given the presence of samples from patients undergoing lung nodule resection surgery, we note that no enrichment for cancer related gene sets was observed in our REACTOME pathway analyses, which argues against bias by cancer diagnosis. With respect to GWAS integration, a more complete set of loci and integration across multiple putative functional variants, along with more uniform interaction measurement methods would provide a deeper understanding of the subtle effects of genetic variants. Although candidate eQTL analyses were included in this study, a future direction would be a genome-wide eQTL analysis^28^,^68^ to examine the direct association between COPD GWAS loci and the expression of genes of interest, including the genes identified in these interactor and network analyses.

By incorporating gene expression, experimental interaction, and network methods, we found significant differences in expression for genes interacting with genes at COPD GWAS loci. Our methodology could be applied to expression data from target tissues in other common diseases. Network analyses highlighted the importance of B cell pathways in COPD, which was recapitulated in the human lung tissue and a mouse smoking model. Replication of previous gene expression results was made possible by careful attention to subject enrollment, including former smokers with severe COPD. Consistent phenotype definitions are likely to be important for other diseases as well.

## Methods

### Study Population

Lung tissue samples were collected from subjects undergoing thoracic surgery for lung transplantation, lung volume reduction surgery or lung nodule resection at three medical centers: Brigham and Women’s Hospital (Boston, MA), St. Elizabeth’s Hospital (Boston, MA) and Temple University Hospital (Philadelphia, PA). Subjects provided written informed consent for use of excess lung tissue for research. IRB approval was obtained at the three centers. All subjects were former smokers, who quit smoking at least one month prior to surgery. Distant normal tissue was used from nodule resection samples. Phenotypes extracted from the medical records included demographics, anthropometrics, smoking history, and spirometry to measure lung function. COPD cases had severe or very severe airflow obstruction (GOLD grades 3-4)^1^. Control smokers had normal lung function. When available, chest computed tomography (CT) scans were retrieved for quantitative image analysis using 3-D Slicer software (www.slicer.org). Emphysema was assessed by the fraction of lung voxels with attenuation less than −950HU (LAA-950) and by the 15^th^ percentile of the lung density histogram (Perc15). Airway disease was quantified by the square root wall area of a hypothetical 10mm internal perimeter airway (SRWA-Pi10)^69^.

### Lung tissue gene expression profiling

Lung tissue samples were snap frozen and stored at −80 °C. RNA and DNA were simultaneously extracted from the homogenized lung tissue using the AllPrep kit (Qiagen, Valenica, CA). RNA quality was assessed on a BioAnalyzer (Agilent, Santa Clara, CA). Gene expression profiling was performed using HumanHT-12 BeadChips (Illumina, San Diego, CA). Quality control was performed using quantile, signal-to-noise, correlation matrix, MA, and principal component analysis (PCA) plots using R statistical software (v 3.2.0) to identify outliers and samples with questionable or low-quality levels, distributions, or associations. In addition, information from other omics data for this cohort was used to cross-check for sample issues during the data cleaning process. This process yielded 151 samples for analysis. There were 32,831 probes retained, after filtering for low variance and percentage of high detection p-values, which denote probes mapped to low expressed genes. This set of probes contains 20,794 unique gene symbol annotations. These data were background corrected, log2 transformed and quantile normalized using the R Bioconductor package lumi^70^. Microarray data has been deposited in Gene Expression Omnibus (GEO accession GSE76925).

### RNA-interference

To find genes that could be targets of *FAM13A*, three *FAM13A* siRNA (Ambion, S19751, S19752 and S19753) were transfected into 16HBE cells. The experiment was controlled using Ambion Silencer Negative Control #1 siRNA (Ambion, 4390843). This control siRNA has no significant sequence similarity to human gene sequences. Cells were collected 48 hours after transfection for RNA extraction, and microarray expression analyses were performed using Illumina HT12 microarrays. Comparisons were made using 2-way ANOVA to determine differentially expressed probes between siRNA-treated and control cells. The intersection of differentially expressed probes from all three comparisons became the final gene list for *FAM13A* siRNA experiments.

### External Datasets

As previously reported, microarray gene expression profiling was performed in human bronchial epithelial cells (Beas-2B) stably infected with four different lentivirus-based shRNAs against *HHIP* and one non-targeting shRNA (control), using the Illumina HT12 gene expression platform^31^. To identify differentially expressed probes after *HHIP* silencing, two linear regression models were used. The set of significantly differentially expressed genes found using both models became the analysis gene list.

Affinity purification followed by mass spectrometry (AP-MS) was performed to identify proteins that bind to either *HHIP*^71^ or *FAM13A*^30^. Briefly, HEK 293 cells were transfected with FLAG-tagged *HHIP* or control vector. Seventy-two hours after transfection, cells were lysed, and protein complexes were immunoprecipitated with an anti-FLAG antibody. *HHIP*-bound proteins were identified by mass spectrometry. Identified proteins were mapped to genes and the control results were subtracted from the experimental results to produce a final gene list for comparison with COPD expression results.

Because the IREB2 protein binds mRNA, RNA immunoprecipitation sequencing (RIP-Seq) was used to identify IREB2 targets^29^. IREB2-RNA complexes were immunoprecipitated from the Beas2B cells and whole transcriptome sequencing was performed. Two replicates in stimulated cells, with deferoxamine (DFO) and DFO-free controls (CTL), were included. In all cases, IREB2 was precipitated, along with IgG (background). The software application HOMER^72^ was used to call peaks accounting for the background using an hg19 read alignment, and four comparisons were made: CTL/IgG, CTL/DFO, DFO/IgG, and DFO/CTL. Peaks common between CTL/IgG and CTL/DFO, CTL/IgG and DFO/IgG, or DFO/IgG and DFO/CTL were mapped to genes and defined the IREB2 target gene list.

Homozygous deletion of *Hhip* in mice is embryonic lethal^73^. Therefore, *Hhip*^+/−^ heterozygous mice (C57BL/6 background) and wild-type littermates were exposed to cigarette smoke or filtered air for six months^32^. RNA was extracted from mouse lungs and profiled in Illumina MouseRef-8 arrays. The differentially expressed genes (FDR < 0.10) were mapped to human orthologs for comparisons to the human lung tissue.

The GeneChip Mouse Gene 1.0 ST Array (Affymetrix) was used to assess gene expression in the *Irp2 (IREB2* gene in humans) knockout and WT mice^29^. The expression difference between the *Irp2* knockout and WT mice was quantified using an unpaired two-tailed t-test. A set of genes was created using the human orthologs from these results.

### Differential gene expression and pathway analyses

Microarray batch effects were identified using MDS plots, and these along with latent effects were addressed by using surrogate variables, obtained via the R/Bioconductor package SVA^74^, as covariates in the linear models. For each expression probe or module eigengene, we fitted a linear regression model to detect association with variables of interest using R statistical software (v 3.2.0). In the microarray data analysis, an empirical Bayes shrinkage method was used to obtain a moderated t-test statistic and its p-value in limma^75^; the log fold change (logFC) values from limma are reported here as fold difference for improved clarity. Adjustment for multiple testing controlled for false discovery rate (FDR). The regression models for each clinical phenotype, including covariates, are provided in Supplemental Table S11. We found no significant association with time since smoking cessation, so this variable was not included as a covariate in subsequent analyses. Using the hypergeometric test in the GeneAnswers package^76^, we examined enrichment of curated gene sets from the Reactome database and from the biological process (BP) category in the gene ontology (GO) database. Hypergenometric tests were performed using the phyper function in the stats R package to observe enrichment of interactors in the differentially expressed gene sets with respect to the background (genes represented in the expression data following QC).

### Weighted Gene Co-expression Analysis (WGCNA)

The WGCNA method was used to identify groups of probes that have similar expression characteristics in the sample population, using the R package WGCNA^43^. Signed networks were built using biweight midcorrelation as the correlation function, and a soft thresholding power of 12. WGCNA produces a set of modules (labeled by color), each containing a set of unique probes. The module eigengenes were used in the regression models^35^ from Supplemental Table S11. Driver or hub genes within each module were identified using previously described methods^35^, and through observation of the degree for each node.

### Genotyping

Genomewide genotyping of extracted lung DNA was performed using the HumanOmni2.5Exome-8 V1.0 BeadChip (Illumina, Inc; San Diego, CA) and quality control assessment was performed using Python and R scripts and PLINK 2^77^. Subject data were excluded after examining missingness, relatedness, sex, and inbreeding. Relatedness was examined using rgGRR and KING^78^, and sex assignment was based on X homozygosity estimates; discordant samples were removed. Principal components were generated for the lung tissue cohort and HapMap3 Public Release #3 panel using EIGENSTRAT^79^, and two additional subjects were subsequently excluded based on genetic ancestry that differed from reported. Manufacturer-identified poorly performing markers, duplicate markers, indels and markers exhibiting ambiguous or failed mapping were removed. Markers with missingness >1%, Hardy-Weinberg equilibrium deviation (p-value < 1e-7) or minor allele frequency <5% were excluded. To account for population stratification, two principal components (based on the Tracy-Widom statistic) for the Caucasian population were retained for use in the statistical analysis.

### Candidate gene expression quantitative trait locus (eQTL) analysis

The eQTL analyses were performed using the 117 Caucasian subjects for whom both expression and genotyping data were available. The R/Bioconductor package Matrix eQTL^80^ was used to perform eQTL analysis to identify associations between genotype and gene expression levels, adjusted for the covariates age, sex, pack-years of smoking and two ancestry principal components. In addition, an iterative method was used to determine the number of expression matrix principal components (PC) to add as covariates to the model to mitigate batch effects. This procedure involves finding the number of PC covariates that produces a maximum number of significant (FDR < 0.05) cis-eQTLs (window of 1 Mb) using genotyping data from chromosome 21 and 22. The maximum was achieved with 13 PCs, and these were included as covariates in the eQTL analyses. Candidate cis-eQTL and trans-eQTL analyses were performed using all probes in the microarray analysis that passed QC and the 108 markers (p-value < 1.0e-5) from a recent GWAS for COPD case-control status^8^ that were available in our genotyping data and annotated by proximity to the three candidate genes of interest. All nominally significant trans-eQTL results (p-value < 0.05) for the three top cis-eQTLs located in each of the three loci (*IREB2* rs2009746, *HHIP* rs11724319, *FAM13A* rs2609255) were used to produce each gene list.

### GWAS results for interactors

The SNPs annotated by proximity to the interactor genes *BCL2, BCL11A, POU2AF1, HMGB1, SERPINE2* and *CD79A* were downloaded from dbSNP. Association p-values for these SNPs were extracted from a published COPD case-control GWAS^8^.

### Ethics Statement

Subjects provided written informed consent for use of excess lung tissue for research. IRB approval was obtained at Partners Healthcare (parent company of Brigham and Women’s Hospital), Temple University and St. Elizabeth’s Hospital. The methods for lung tissue research were carried out in accordance with the relevant guidelines. The mouse protocols were approved by the Institutional Animal Care and Use Committee, Harvard Medical School, and the methods were carried out in accordance with the relevant guidelines, as detailed in previous publications^29^,^32^,^71^.

## Additional Information

**How to cite this article:** Morrow, J. D. *et al*. Functional interactors of three genome-wide association study genes are differentially expressed in severe chronic obstructive pulmonary disease lung tissue. *Sci. Rep.* **7**, 44232; doi: 10.1038/srep44232 (2017).

**Publisher's note:** Springer Nature remains neutral with regard to jurisdictional claims in published maps and institutional affiliations.

## Supplementary Material

Supplementary Material

Supplementary Table S2

## Figures and Tables

**Table 1:** Top 20 genes differentially expressed in COPD vs. control.

Probe name	Log Fold Difference^*^	p-value	FDR q-value	Gene symbol
ILMN_1676938	−0.9164	5.40E-010	1.77E-005	*HMGB1*
ILMN_3187508	−0.6609	1.70E-009	2.79E-005	*FLJ40504*
ILMN_2050921	−0.6510	3.16E-009	3.46E-005	*C3orf78*
ILMN_2398474	−0.7957	2.11E-008	1.73E-004	*RAP1B*
ILMN_3245015	−0.6185	3.18E-008	2.09E-004	*LOC440563*
ILMN_1665333	−0.7186	6.52E-008	3.57E-004	*SUMO2*
ILMN_1705330	−0.7185	8.61E-008	4.04E-004	*CDC42*
ILMN_1656898	−0.7223	1.31E-007	5.37E-004	*PTCD1*
ILMN_3266294	−0.6043	1.66E-007	6.05E-004	*SREK1IP1*
ILMN_1696031	−0.5292	1.99E-007	6.22E-004	*C15orf21*
ILMN_1691485	−0.6776	2.08E-007	6.22E-004	*GTF2H2*
ILMN_3191922	−0.8825	2.28E-007	6.24E-004	*FLJ46111*
ILMN_2068122	−0.6563	3.42E-007	8.63E-004	*TMEM65*
ILMN_3239871	−0.5563	4.85E-007	1.07E-003	*ARPP19*
ILMN_3219808	−0.5897	4.90E-007	1.07E-003	*HNRNPA1*
ILMN_1716246	0.8208	5.94E-007	1.22E-003	*FRZB*
ILMN_3183789	−0.3577	7.60E-007	1.47E-003	*UQCRFS1*
ILMN_1799837	−0.6066	1.07E-006	1.95E-003	*POTEM*
ILMN_2141398	−0.6715	1.32E-006	2.24E-003	*VEZT*
ILMN_1674399	0.3467	1.40E-006	2.24E-003	*ZNF143*

*Reference group is controls: positive log fold difference corresponds to higher expression in cases.

**Table 2:** Differential expression results for putative genes at previously identified genome-wide significant COPD and emphysema GWAS loci (probe with highest ranking result shown).

Gene	Probe	P-value	FDR q-value	Log fold difference^*^
*MMP12*	ILMN_2073758	0.02	0.30	0.91
*HHIP*	ILMN_1675453	0.04	0.42	0.41
*AGER*	ILMN_1729777	0.11	0.59	−0.31
*IREB2*	ILMN_1726554	0.12	0.60	0.21
*DLC1*	ILMN_1698020	0.16	0.66	−0.24
*CHRNA5*	ILMN_1770044	0.19	0.67	0.12
*RAB4B*	ILMN_1761896	0.33	0.80	0.096
*FAM13A*	ILMN_1800267	0.34	0.80	−0.09
*CHRNA3*	ILMN_2154157	0.53	0.89	0.13
*RIN3*	ILMN_1731736	0.64	0.93	0.07
*TGFB2*	ILMN_1812526	0.89	0.98	−0.03

*Reference group is controls: positive log fold difference corresponds to higher expression in cases.

**Table 3:** Enrichment of functional interactors of COPD GWAS genes in the gene expression results (differentially expressed genes with FDR < 0.05).

Experiment	Number of genes or proteins*	Overlap with 204 COPD genes	Enrichment p-value for COPD genes	Overlap with 1,366 FEV1% predicted genes	Enrichment p-value for FEV1% predicted genes	Overlap with 1,429 FEV1/FVC genes	Enrichment p-value for FEV1/FVC genes
*IREB2* RIP-seq	4008	64	2.4e-5	359	3.6e-11	400	<1.0e-12
*HHIP* affinity purification	216	5	0.062	34	1.8e-6	45	1.6e-11
*FAM13A* affinity purification	97	0	1	8	0.31	8	0.35
*Hhip*^+/+^ vs. *Hhip*^+/−^ mouse, 6 mo smoke	492	15	0.00011	60	2.7e-6	61	5.4e-6
*Hhip* mouse, genotype x smoke interaction	549	12	0.0082	46	0.054	46	0.095
*IREB2 Irp2*^+/+^ vs. *Irp2*^−/−^ mouse knockout	31	0	1	3	0.33	3	0.36
*HHIP* shRNA knockdown	266	3	0.49	20	0.30	19	0.46
*FAM13A* siRNA knockdown	598	13	0.0063	54	0.011	61	0.0013
*IREB2* trans-eQTLs at p < 0.05	1612	31	0.00023	171	1.4e-10	159	1.7e-6
*HHIP* trans-eQTLs at p < 0.05	1560	21	0.087	143	2.4e-05	156	9.4e-7
*FAM13A* trans-eQTL at p < 0.05	1753	38	2.7e-06	209	<1.0e-12	194	8.7e-12

*Unique gene annotations found in the final expression data.
