# Supplementary material for: Functional interactors of three genome-wide association study genes are differentially expressed in severe chronic obstructive pulmonary disease lung tissue
Source: Sci Rep. 2017 Mar 13;7:44232. doi: 10.1038/srep44232 (PMC5347019; doi:10.1038/srep44232)
Supplement: Supplementary Material [file srep44232-s1.pdf]

Functional interactors of three genome-wide association study genes are differentially expressed in severe chronic obstructive pulmonary disease lung tissue

Jarrett D. Morrow[1], Xiaobo Zhou[1], Taotao Lao[1], Zhiqiang Jiang[1], Dawn L. DeMeo[1,2], Michael H. Cho[1,2], Weiliang Qiu[1], Suzanne Cloonan[3], Victor Pinto-Plata[4], Bartholome Celli[2], Nathaniel Marchetti[5], Gerard J. Criner[5], Raphael Bueno[6], George R. Washko[2], Kimberly Glass[1], John Quackenbush[7], Augustine M. K. Choi[3], Edwin K. Silverman[1,2], Craig P. Hersh[1,2,#]

1. Channing Division of Network Medicine, Brigham and Women's Hospital, Boston, MA

2. Division of Pulmonary and Critical Care Medicine, Brigham and Women's Hospital, Boston, MA

3. Department of Medicine, New York Presbyterian/Weill Cornell Medical Center, New York, NY

4. Department of Critical Care Medicine and Pulmonary Disease, Baystate Medical Center, Springfield, MA

5. Division of Pulmonary and Critical Care Medicine, Temple University, Philadelphia, PA

6. Division of Thoracic Surgery, Brigham and Women's Hospital, Boston, MA

7. Department of Biostatistics and Computational Biology, Dana-Farber Cancer Institute, Boston, MA

# Corresponding Author:

Craig P. Hersh

Channing Division of Network Medicine

Brigham and Women's Hospital

181 Longwood Avenue, Boston, MA 02115

Phone: 617-525-0729, Fax: 617-525-0958

craig.hersh@channing.harvard.edu

S1 Table. Demographics of study subjects with expression data

**Demographics**	**COPD cases n=111**	**Control smokers n=40**	**P-value**
Age (years)	63.3 (± 6.6)	65.7 (± 9.0)	0.12
Gender (female)	59 (53.2%)	25 (62.5%)	0.36
Race			0.91
African American	18 (16.2%)	5 (12.5%)	
White	90 (81.1%)	34 (85.0%)	
Other	3 (2.7%)	1 (2.5%)	
Current Smokers	0%	0%	NA
Smoking History (pack-years)	61.3 (± 26.3)	33.6 (± 21.0)	<0.0001
Time since quitting, months	81.1 (± 85.0)	187.1 (± 144.8)	<0.0001
FEV1 % predicted	26.5 (± 9.4)	98.7 (± 12.5)	<0.0001
FEV1/FVC	0.3 (± 0.1)	0.8 (± 0.1)	<0.0001
Body Mass Index	25.6 (±4.6)	28.2 (±5.8)	0.02
CT emphysema: Low attenuation areas at -950 HU (85 cases, 18 controls)	34% (±14%)	5% (±6%)	<0.0001
CT emphysema: 15th percentile of the lung density histogram (85 cases, 18 controls)	-975 (±24.0)	-910.2 (±35.1)	<0.0001
SRWA-Pi10 (53 cases, 11 controls)	4.3 (±0.3)	4.0 (±0.4)	0.08

Abbreviations: FEV1=forced expiratory volume in 1 sec; FVC= forced vital capacity; CT=computed tomography; SRWA-Pi10=square root wall area of a hypothetical airway with 10mm internal perimeter

S3 Table: Overview of gene expression results

**Phenotype variable predictor**	**Significant probes (FDR<0.05)**
COPD	214
FEV1 % predicted	1,556
FEV1 % predicted (cases only)	6
FEV1/FVC	1,689
FEV1/FVC (cases only)	57
CT emphysema: Low attenuation areas at -950 HU	143
CT emphysema: 15th percentile of the lung density histogram	223
SRWA-Pi10	0

Abbreviations: FEV1=forced expiratory volume in 1 sec; FVC= forced vital capacity;
CT=computed tomography;
SRWA-Pi10=square root wall area of a hypothetical airway with 10mm internal perimeter

S4 Table. Top pathway enrichment results for the 214 probes (204 genes) associated with COPD (FDR<0.05)

**GO description**	**ID**	**Genes in Category**	**p value**	**FDR q value**
cellular protein modification process	GO:6464	49	1.13E-004	0.017
apoptotic process	GO:6915	34	3.10E-005	0.012
phosphorylation	GO:16310	31	1.58E-003	0.045
regulation of apoptotic process	GO:42981	25	7.07E-004	0.034
regulation of protein modification process	GO:31399	24	1.14E-003	0.041
protein phosphorylation	GO:6468	24	4.39E-003	0.072
peptidyl-amino acid modification	GO:18193	22	3.41E-004	0.027
regulation of phosphorylation	GO:42325	21	6.29E-003	0.086
generation of neurons	GO:48699	21	1.04E-002	0.097
positive regulation of protein modification process	GO:31401	20	4.07E-004	0.028
cytoplasmic transport	GO:16482	19	2.33E-003	0.05
positive regulation of phosphate metabolic process	GO:45937	18	3.88E-003	0.066
regulation of protein phosphorylation	GO:1932	18	7.92E-003	0.091
protein modification by small protein conjugation or removal	GO:70647	17	1.44E-003	0.043
positive regulation of phosphorylation	GO:42327	17	2.52E-003	0.052
intracellular protein transport	GO:6886	17	8.17E-003	0.091
**Reactome category**	**ID**	**Genes in Category**	**p value**	**FDR q value**
Regulation of actin dynamics for phagocytic cup formation	2029482	6	6.72E-005	0.035
Fcgamma receptor (FCGR) dependent phagocytosis	2029480	6	3.91E-004	0.1

S5 Table. Lists of intersecting genes represented in the columns of S1 Figure.

**Figure S1 column**	**Intersecting genes**
1	*CDC42, PSMC6, OAZ1, FBXO5, SENP6, EXOC8, YPEL5, UBQLN1, HTR2B, SERPINE2, GCC2, RAC1, SDCBP, SKP2, GOLGA7, UBASH3B, TAGLN2, FRZB, TMEM65, BMS1, PPIB, UQCRFS1, CXXC5, BPGM, EIF3F, NQO1, SELT, KIAA1279, ARFGAP3*
2	*GYPE, TEX2, H3F3C, ABCA11P, DACH1, CARD16, MACC1, MRPL47, SYNE1, SMN2, ZCCHC10, HSP90AB4P, GTF2H2, PGGT1B, NNT*
3	*ERCC6, CLIC1, PPIA, SEPT6, CCL19, EMP2, RBMS1, PGAM4, S100A10, ARAP2*
4	*RBM33, PAQR8, CILP, CCT6P1, IRX6, CIT, ZNF101, PI4K2B*
5	*POU2AF1, PLA2G7, RANBP9, DTX1*
6	*XRCC4, PHKB, VEZT, PHF14*
7	*TPM3*
8	*CAND2*
9	*ABL1, CD79A, FBXW7, FNTA, SPCS2, EEF1B2, VPS35, RPSA, HMGB1, P2RY14, PPP3CB*
10	*C6orf62, ADH5, SUMO2, C11orf58, VEGFA, SNRPG*
11	*BCL11A, CXCL12, E2F6, FCGR3A*
12	*TM4SF19, DNTTIP2, HSPA5, HSPA13*
13	*SYF2, ARPP19, BUB3, NUDT21*
14	*KRT18, SLC45A2, SLC25A12*
15	*MYO5B, PGK1*
16	*BCLAF1*
17	*LOC401397*
18	*AKAP2*
19	*PDPK1*
20	*KRT8*
21	*HSP90AA1, VDAC1*
22	*CXCR5*
23	*CLEC2D*

24	*TOP1*
25	*SOCS4*
26	*EDN1*
27	*DNAJB14*
28	*BCL2*
29	*BLK*
30	*SELM*
31	*ADD3*
32	*HNRNPA1*
33	*ELMO1*
34	*LIMS1*
35	*ARL4A*
36	*CCBL2*

S6 Table. Summary of WGCNA modules (modules of interest highlighted)

Module name	Minimum module self correlation	Mean module self correlation	SEM module self correlation	Module size
black	0.1739	0.6958	0.0069	911
brown	0.0080	0.6885	0.0030	4505
red	0.1767	0.7047	0.0053	1115
greenyellow	0.4013	0.6791	0.0060	273
midnightblue	0.5756	0.7880	0.0090	79
turquoise	0.0110	0.5277	0.0019	5518
yellow	0.0261	0.5869	0.0033	2845
lightcyan	0.4093	0.7497	0.0292	30
pink	0.2736	0.6214	0.0055	495
salmon	0.3093	0.7392	0.0102	131
blue	0.0336	0.5623	0.0025	4603
purple	0.3392	0.6841	0.0051	486
cyan	0.3967	0.7219	0.0118	90
magenta	0.3144	0.6452	0.0042	495
green	0.0289	0.5358	0.0034	2543
tan	0.2853	0.6158	0.0129	197
grey*	0.0001	0.2152	0.0017	8515

*grey module is a collection of probes with low correlation across all modules and is therefore not included in the analysis

S7 Table. Cyan module membership (putative hubs highlighted)

Probe	Gene Significance (q-value)	Module Membership (correlation with eigengene)	Gene symbol	Differentially expressed (FDR<0.1) in mouse *Hhip*$^{+/+}$ vs. *Hhip*$^{+/-}$ 6 month smoke experiment[32]	Associated with regional emphysema severity in Campbell et al. study (FDR<0.1)[10]	Associated with emphysema vs. bronchiolitis in lung tissue in Faner et al. study[44]
ILMN_1734878	0.0315	0.9291	*CD79A*		Yes	
ILMN_1811049	0.0277	0.9148	*POU2AF1*	Yes		Yes
ILMN_2401714	0.0177	0.6307	*MS4A1*			Yes
ILMN_1791329	0.0385	0.7830	*FCRL2*			
ILMN_1668277	0.0399	0.8413	*BLK*	Yes		
ILMN_2342271	0.0399	0.5205	*BCL11A*	Yes	Yes	
ILMN_2337928	0.0499	0.7747	*CXCR5*	Yes		
ILMN_1782704	0.0548	0.8593	*CD19*	Yes		
ILMN_1873838	0.0677	0.6375	*IGKC*			Yes
ILMN_2369666	0.0860	0.6814	*CR2*		Yes	Yes
ILMN_1708798	0.0943	0.7488	*EAF2*	Yes		
ILMN_2414762	0.0951	0.8305	*TLR10*			
ILMN_1674228	0.1065	0.7828	*abparts**			
ILMN_1691071	0.1163	0.8791	*FCRLA*	Yes	Yes	
ILMN_2393765	0.1164	0.7392	*IGLL1*			
ILMN_1719905	0.1182	0.8361	*TLR10*			
ILMN_2193233	0.1324	0.8565	*MZB1*			Yes
ILMN_1684445	0.1332	0.7497	*FCRL5*			Yes
ILMN_1804601	0.1342	0.7993	*DKFZp686O16217*			
ILMN_1711988	0.1374	0.3967	*KCNK12*			
ILMN_1776939	0.1427	0.6432	*MS4A1*			Yes
ILMN_1699214	0.1559	0.8390	*abparts**			
ILMN_1811238	0.1602	0.4916	*ALPK2*			
ILMN_1739508	0.1648	0.8318	*abparts**			
ILMN_3240375	0.1665	0.7672	*abparts**			
ILMN_1680274	0.1731	0.7781	*abparts**			
ILMN_1700147	0.1778	0.8365	*VPREB3*			
ILMN_1759075	0.1821	0.8549	*TNFRSF13B*			
ILMN_1659227	0.1969	0.9235	*CD79A*		Yes	
ILMN_1804339	0.1977	0.8408	*CAMK1G*			
ILMN_1707491	0.1977	0.8470	*FAM30A*			
ILMN_1768016	0.2123	0.8708	*TNFRSF17*	Yes		Yes

ILMN_1785439	0.2326	0.7982	*CD79B*	Yes	Yes	
ILMN_1711514	0.2503	0.6311	*COCH*			
ILMN_1739726	0.2509	0.8058	*JSRP1*			
ILMN_1688959	0.2555	0.8473	*CD27*	Yes		Yes
ILMN_1652199	0.2629	0.8368	*abparts**			
ILMN_2298366	0.2825	0.6390	*TLR10*			
ILMN_2366212	0.2825	0.7715	*CD79B*	Yes	Yes	
ILMN_1802100	0.2838	0.5599	*ADAM28*			
ILMN_1679798	0.2899	0.6038	*TLR9*			
ILMN_1731111	0.2922	0.5239	*DERL3*	Yes	Yes	Yes
ILMN_1700428	0.2939	0.7724	*HLA-DOB*	Yes		
ILMN_1718552	0.2945	0.7078	*CXCL13*		Yes	Yes
ILMN_1676003	0.3196	0.8910	*PNOC*			
ILMN_3247998	0.3205	0.7472	*STAP1*			
ILMN_1664631	0.3256	0.6002	*ADAM28*			
ILMN_2105441	0.3267	0.7638	*IGJ*			Yes
ILMN_1731742	0.3314	0.7403	*TNFRSF13C*	Yes		
ILMN_1707448	0.3360	0.8136	*CDK12*			
ILMN_2073202	0.3447	0.6831	*MEI1*			
ILMN_2099528	0.3458	0.7638	*BTLA*	Yes		
ILMN_2214355	0.3554	0.6887	*RAB30*			
ILMN_1684724	0.3570	0.6742	*CR2*		Yes	Yes
ILMN_1759312	0.3580	0.7688	*AMPD1*		Yes	
ILMN_2403965	0.3681	0.7066	*TXNDC5*			
ILMN_1695891	0.3703	0.7158	*abparts**			
ILMN_1659800	0.3773	0.5470	*BCL11A*	Yes	Yes	
ILMN_1748283	0.3892	0.8224	*PIM2*			Yes
ILMN_1749502	0.3976	0.7694	*ZNF215*			
ILMN_1713759	0.4292	0.6289	*UBE2J1*			
ILMN_1743797	0.4304	0.7236	*abparts**			
ILMN_1777971	0.4697	0.6619	*RNF126P1*			
ILMN_1810254	0.4849	0.8117	*MEI1*			
ILMN_1769782	0.5019	0.6764	*LAX1*	Yes		
ILMN_1710017	0.5397	0.7897	*CD79B*	Yes	Yes	
ILMN_1787345	0.5512	0.8560	*FKBP11*			
ILMN_1729198	0.5564	0.6374	*abparts**			
ILMN_1810374	0.5610	0.7745	*TMEM156*			
ILMN_2206722	0.5752	0.6824	*FER1L4*			
ILMN_1653579	0.5768	0.6445	*abparts**			

ILMN_3212395	0.5815	0.7890	*IGH*			
ILMN_1678859	0.6029	0.6176	*abparts**			
ILMN_1788108	0.6268	0.7740	*TXNDC5*			
ILMN_1658426	0.6273	0.6915	*WNT10A*			
ILMN_3187535	0.6498	0.6201	*FAM30A*			
ILMN_3247797	0.6510	0.6073	*abparts**			
ILMN_1813703	0.6541	0.6466	*RALGPS2*	Yes	Yes	
ILMN_1681301	0.6624	0.6719	*AIM2*			Yes
ILMN_1806754	0.6906	0.5530	*GLDC*	Yes		
ILMN_1749362	0.7112	0.5387	*CD28*			
ILMN_1769082	0.7143	0.6205	*TXNDC5*			
ILMN_3307791	0.7247	0.5610	*FAM69A*			
ILMN_1696705	0.7905	0.5272	*<NA>*			
ILMN_2233783	0.8128	0.7426	*CD38*			Yes
ILMN_1799669	0.8213	0.6883	*PSORS1C1*			
ILMN_1651415	0.8222	0.7224	*DERL3*	Yes	Yes	Yes
ILMN_1652366	0.8955	0.5934	*NLRP7*			
ILMN_3243573	0.9788	0.5583	*abparts**			
ILMN_1713266	0.9847	0.6553	*FAM46C*			
*probe mapping to hypervariable antibody regions						

S8 Table. Top 109 Brown module members with nominal Gene Significance (p-value<0.05) sorted by driver metrics (putative driver highlighted).

**Probe**	**Gene Significance (p-value)**	**Module Membership**	**Gene symbol**
ILMN_1676938	1.77E-005	0.8357	*HMGB1*
ILMN_3187508	2.79E-005	0.5224	*FLJ40504*
ILMN_2050921	3.46E-005	0.8691	*C3orf78 (SMIM4)*
ILMN_2398474	0.0001734	0.8756	*RAP1B*
ILMN_3245015	0.0002088	0.8362	*LOC440563*
ILMN_1665333	0.0003569	0.5896	*SUMO2*
ILMN_1705330	0.000404	0.8291	*CDC42*
ILMN_1656898	0.0005366	0.8440	*PTCD1*
ILMN_3266294	0.0006049	0.6486	*SREK1IP1*
ILMN_1696031	0.000622	0.7596	*C15orf21*
ILMN_1691485	0.000622	0.9047	*GTF2H2*
ILMN_3191922	0.0006237	0.7170	*FLJ46111*
ILMN_2068122	0.0008625	0.8763	*TMEM65*
ILMN_3239871	0.001072	0.8138	*ARPP19*
ILMN_3219808	0.001072	0.8429	*HNRNPA1*
ILMN_3183789	0.001467	0.5417	*UQCRFS1*
ILMN_1799837	0.001945	0.8195	*POTEM*
ILMN_2141398	0.002241	0.7662	*VEZT*
ILMN_2133146	0.002241	0.9234	*NUDT21*
ILMN_1680887	0.002531	0.6139	*CLIC1*
ILMN_3251365	0.002555	0.8500	*PGGT1B*
ILMN_1711053	0.002555	0.6945	*BBS5*
ILMN_1695872	0.002555	0.9327	*CDC42*
ILMN_3284222	0.002596	0.9094	*EMP2*
ILMN_2168866	0.002836	0.8063	*C6orf62*
ILMN_3178307	0.002836	0.7763	*KRT18*
ILMN_1681160	0.003019	0.9066	*CCT6P1*
ILMN_3201895	0.003288	0.8183	*TAF9*
ILMN_2102515	0.003633	0.6678	*PGAM4*

ILMN_1718001	0.003677	0.7394	*PHKB*
ILMN_1709360	0.004273	0.7581	*ARAP2*
ILMN_1783303	0.005206	0.8818	*HSP90AB4P*
ILMN_1652209	0.005421	0.9106	*BMS1*
ILMN_1762808	0.005421	0.9438	*RAC1*
ILMN_2372773	0.005617	0.7647	*TANK*
ILMN_1766753	0.006712	0.9335	*RBMS1*
ILMN_2167709	0.007051	0.6860	*NIPSNAP3A*
ILMN_2407605	0.007418	0.6921	*SLX1A*
ILMN_3246845	0.007747	0.6926	*VPS35*
ILMN_3195815	0.008009	0.9020	*HMGB1*
ILMN_1812163	0.009411	0.5508	*GYPE*
ILMN_2245180	0.009411	0.7795	*BUB3*
ILMN_2211583	0.009411	0.9642	*KIAA1279*
ILMN_1842286	0.009411	0.9159	*AV740359*
ILMN_3188174	0.009499	0.8307	*CLEC2D*
ILMN_2130003	0.009499	0.9054	*CCBL2*
ILMN_3256445	0.0105	0.8845	*SNRPG*
ILMN_2147471	0.0105	0.9561	*PI4K2B*
ILMN_2399571	0.0105	0.9453	*CBY1*
ILMN_1654043	0.0105	0.7842	*NBEAL1*
ILMN_3249435	0.0105	0.8262	UBASH3B
ILMN_1764163	0.01142	0.9275	TPM3
ILMN_3302139	0.01177	0.9093	C15orf21
ILMN_1703622	0.01177	0.9346	PPIB
ILMN_1757084	0.01177	0.9344	ADH5
ILMN_2352926	0.01248	0.8199	BPGM
ILMN_1813131	0.01282	0.8502	KRT8
ILMN_2096759	0.01289	0.9551	PSMC6
ILMN_3201975	0.01333	0.9750	PPIA
ILMN_1759396	0.0136	0.8968	NNT
ILMN_1791933	0.01388	0.7863	CCDC82
ILMN_2237474	0.01507	0.7548	TBC1D8B
ILMN_2116811	0.01507	0.8778	C7orf70

ILMN_1654195	0.01585	0.7779	GCC2
ILMN_2086952	0.01619	0.8338	TOP1
ILMN_2354953	0.01669	0.7680	NQO1
ILMN_2184262	0.0173	0.7930	OAS3
ILMN_2099798	0.01759	0.9535	LYPLA1
ILMN_3252936	0.01771	0.8724	KRT18
ILMN_2141157	0.01771	0.9398	RANBP9
ILMN_2213680	0.02059	0.8022	ZCCHC10
ILMN_2294762	0.02059	0.8736	AMY1A
ILMN_1800575	0.02171	0.7692	LIMS1
ILMN_1764036	0.02178	0.9015	P2RY14
ILMN_1734149	0.02425	0.8498	RPSAP52
ILMN_2386053	0.02562	0.8564	DACH1
ILMN_1757011	0.02699	0.8491	LY75
ILMN_2287296	0.02767	0.8386	SYF2
ILMN_1811029	0.02767	0.8218	TLK1
ILMN_3249064	0.02795	0.7918	LOC653545
ILMN_1807945	0.02827	0.9598	ANP32A
ILMN_1773174	0.02884	0.8074	PDPK1
ILMN_2118910	0.02987	0.9545	TPRKB
ILMN_2188959	0.02996	0.8325	ACOT2
ILMN_1871688	0.03172	0.7759	AK093898
ILMN_1687036	0.03207	0.9712	MRPL47
ILMN_3200430	0.03323	0.8377	GOLGA7
ILMN_3258027	0.03426	0.9560	LOC401397
ILMN_3291921	0.03505	0.9366	C11orf58
ILMN_2413264	0.03556	0.9129	SOCS4
ILMN_2379695	0.03675	0.8581	XRCC4
ILMN_1753663	0.03702	0.9256	ARL4A
ILMN_2264916	0.03739	0.8382	EDNRB
ILMN_3205924	0.03853	0.9396	EIF3F
ILMN_2057389	0.03853	0.9189	PEX3
ILMN_2054236	0.03853	0.9303	SENP6
ILMN_1777301	0.03853	0.8017	ARL13B

ILMN_3305993	0.03994	0.9362	OAZ1
ILMN_1762330	0.03994	0.8561	C1D
ILMN_2227368	0.03994	0.9364	SELT
ILMN_2083818	0.04012	0.9581	EXOC8
ILMN_2393060	0.04152	0.8528	PPIL3
ILMN_3191030	0.0418	0.9276	SDCBP
ILMN_3230286	0.04188	0.9406	BCLAF1
ILMN_3201658	0.04219	0.8176	VDAC1
ILMN_1708345	0.04383	0.9503	DNTTIP2
ILMN_2121189	0.0458	0.8426	METTL18
ILMN_3252556	0.04645	0.9199	CARD16
ILMN_2401770	0.04655	0.8887	PHF14

S9 Table. Pathway enrichment results for cyan module.

GO description	ID	genes in category	p value	FDR q value
immune response-regulating cell surface receptor signaling pathway	2768	12	3.33E-010	7.89E-008
B cell proliferation	42100	8	1.33E-010	6.30E-008
antigen receptor-mediated signaling pathway	50851	8	9.25E-009	1.10E-006
regulation of B cell activation	50864	7	1.44E-008	1.37E-006
B cell receptor signaling pathway	50853	6	1.64E-009	2.59E-007
regulation of lymphocyte proliferation	50670	6	1.20E-005	7.34E-004
regulation of mononuclear cell proliferation	32944	6	1.24E-005	7.34E-004
T cell aggregation	70489	6	1.19E-003	2.89E-002
lymphocyte aggregation	71593	6	1.22E-003	2.89E-002
leukocyte aggregation	70486	6	1.31E-003	2.96E-002
leukocyte differentiation	2521	6	1.61E-003	3.46E-002
regulation of B cell proliferation	30888	5	5.47E-007	4.32E-005
immunoglobulin mediated immune response	16064	5	2.49E-005	1.31E-003
B cell mediated immunity	19724	5	2.81E-005	1.33E-003
regulation of protein secretion	50708	5	3.22E-003	4.92E-002
positive regulation of B cell activation	50871	4	3.21E-005	1.38E-003
B cell differentiation	30183	4	2.51E-004	9.91E-003
regulation of calcium ion transport	51924	4	2.13E-003	4.21E-002
**Reactome category**	**ID**	**genes in category**	**p value**	**FDR q value**
Immune System	168256	10	1.02E-005	4.84E-004
Adaptive Immune System	1280218	7	3.07E-005	9.72E-004
Signaling by the B Cell Receptor (BCR)	983705	5	5.57E-005	1.32E-003
Innate Immune System	168249	5	8.49E-003	7.55E-002
Antigen activates B Cell Receptor (BCR) leading to generation of second messengers	983695	4	7.15E-007	6.79E-005
Peptide ligand-binding receptors	375276	3	9.53E-003	7.55E-002
G alpha (i) signalling events	418594	3	1.13E-002	8.22E-002

S10 Table. Pathway enrichment results for brown module.

**GO description**	**ID**	**genes in category**	**p value**	**FDR q value**
RNA biosynthetic process	32774	772	2.97E-028	1.50E-025
regulation of cellular macromolecule biosynthetic process	2000112	750	5.33E-021	1.35E-018
nucleic acid-templated transcription	97659	705	2.22E-018	5.04E-016
transcription, DNA-templated	6351	701	2.72E-018	5.88E-016
regulation of RNA biosynthetic process	2001141	687	1.72E-017	3.12E-015
regulation of nucleic acid-templated transcription	1903506	686	6.09E-018	1.26E-015
regulation of transcription, DNA-templated	6355	680	1.33E-017	2.62E-015
cellular protein modification process	6464	651	8.94E-020	2.14E-017
transcription from RNA polymerase II promoter	6366	357	6.62E-009	6.52E-007
phosphorylation	16310	357	1.35E-005	6.76E-004
apoptotic process	6915	348	5.97E-008	5.43E-006
regulation of transcription from RNA polymerase II promoter	6357	320	1.11E-006	7.01E-005
protein phosphorylation	6468	278	1.91E-005	9.15E-004
regulation of protein modification process	31399	270	5.03E-008	4.67E-006
cytoplasmic transport	16482	269	2.95E-023	9.57E-021
regulation of apoptotic process	42981	269	7.03E-007	4.90E-005
regulation of phosphate metabolic process	19220	266	7.25E-004	1.84E-002
positive regulation of RNA biosynthetic process	1902680	254	5.93E-007	4.28E-005
positive regulation of transcription, DNA-templated	45893	249	5.81E-007	4.26E-005
positive regulation of nucleic acid-templated transcription	1903508	249	5.81E-007	4.26E-005
**Reactome category**	**ID**	**genes in category**	**p value**	**FDR q value**
Metabolism	1430728	270	2.75E-009	1.23E-007
Gene Expression	74160	253	8.07E-040	1.09E-036
Metabolism of proteins	392499	194	3.25E-030	2.19E-028
Immune System	168256	193	2.96E-010	1.38E-008
Disease	1643685	183	2.27E-023	1.39E-021
Infectious disease	5663205	124	3.28E-025	2.11E-023
Cell Cycle	1640170	124	8.93E-013	4.46E-011
Innate Immune System	168249	108	1.05E-004	1.77E-003
Cell Cycle, Mitotic	69278	105	7.93E-012	3.82E-010
Adaptive Immune System	1280218	88	4.29E-005	8.76E-004
Metabolism of lipids and lipoproteins	556833	86	1.20E-002	6.57E-002

Translation	72766	85	1.19E-037	2.30E-035
Signaling by Rho GTPases	194315	81	3.45E-007	1.33E-005
Hemostasis	109582	79	6.91E-003	4.47E-002
Cap-dependent Translation Initiation	72737	75	7.93E-038	1.78E-035
Eukaryotic Translation Initiation	72613	75	7.93E-038	1.78E-035
3' -UTR-mediated translational regulation	157279	73	7.67E-039	3.44E-036
L13a-mediated translational silencing of Ceruloplasmin expression	156827	73	7.67E-039	3.44E-036
GTP hydrolysis and joining of the 60S ribosomal subunit	72706	73	2.03E-038	6.84E-036

S11 Table. Regression models for association with outcomes of interest

Model #	Variable of interest	Model
1	COPD	EXP ~ **COPD** + age + sex + race + pack-years + surrogate_variables
2	FEV1 % predicted	EXP ~ **FEV1.PP** + age + sex + race + pack-years + surrogate_variables
3	FEV1/FVC	EXP ~ **FEV1FVC** + age + sex + race + pack-years + surrogate_variables
4	CT emphysema: Low attenuation areas at -950 HU	EXP ~ **LAA950**+ age + sex + race + BMI + pack-years + surrogate_variables
5	CT emphysema: 15th percentile of the lung density histogram	EXP ~ **perc15** + age + sex + race + BMI + pack-years + surrogate_variables
6	SRWA-Pi10	EXP ~ **Pi10** + age + sex + race + pack-years + surrogate_variables
Abbreviations: FEV1=forced expiratory volume in 1 sec; FVC= forced vital capacity; CT=computed tomography; SRWA-Pi10=square root wall area of a hypothetical airway with 10mm internal perimeter, EXP = expression values		

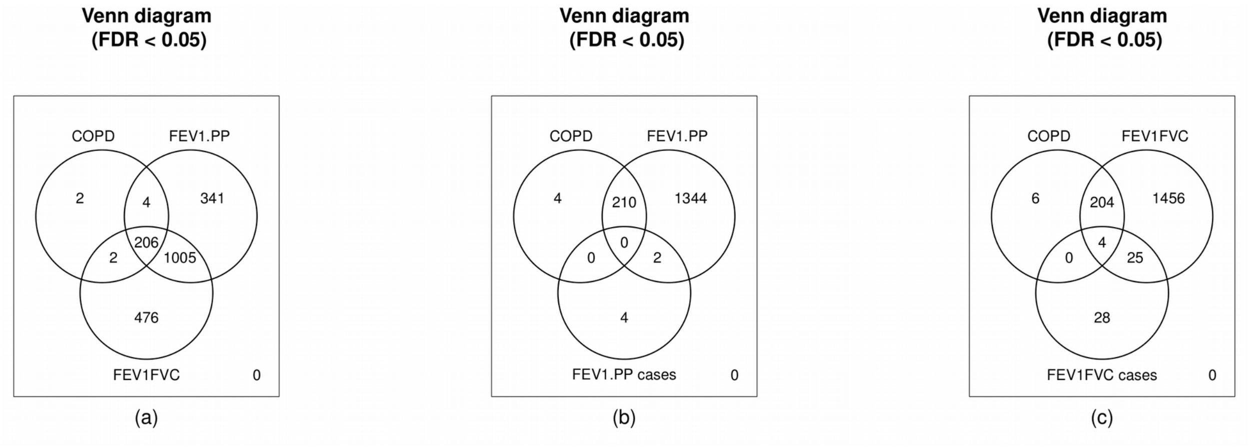

S1 Figure. Venn diagrams of the overlap between the significant differential expression results: (a) case-control, FEV_1 %predicted and FEV_1/FVC ratio significant probes, (b) case-control, FEV_1 %predicted and FEV_1 %predicted (cases only), (c) case-control, FEV_1/FVC ratio and FEV_1/FVC ratio (cases only)

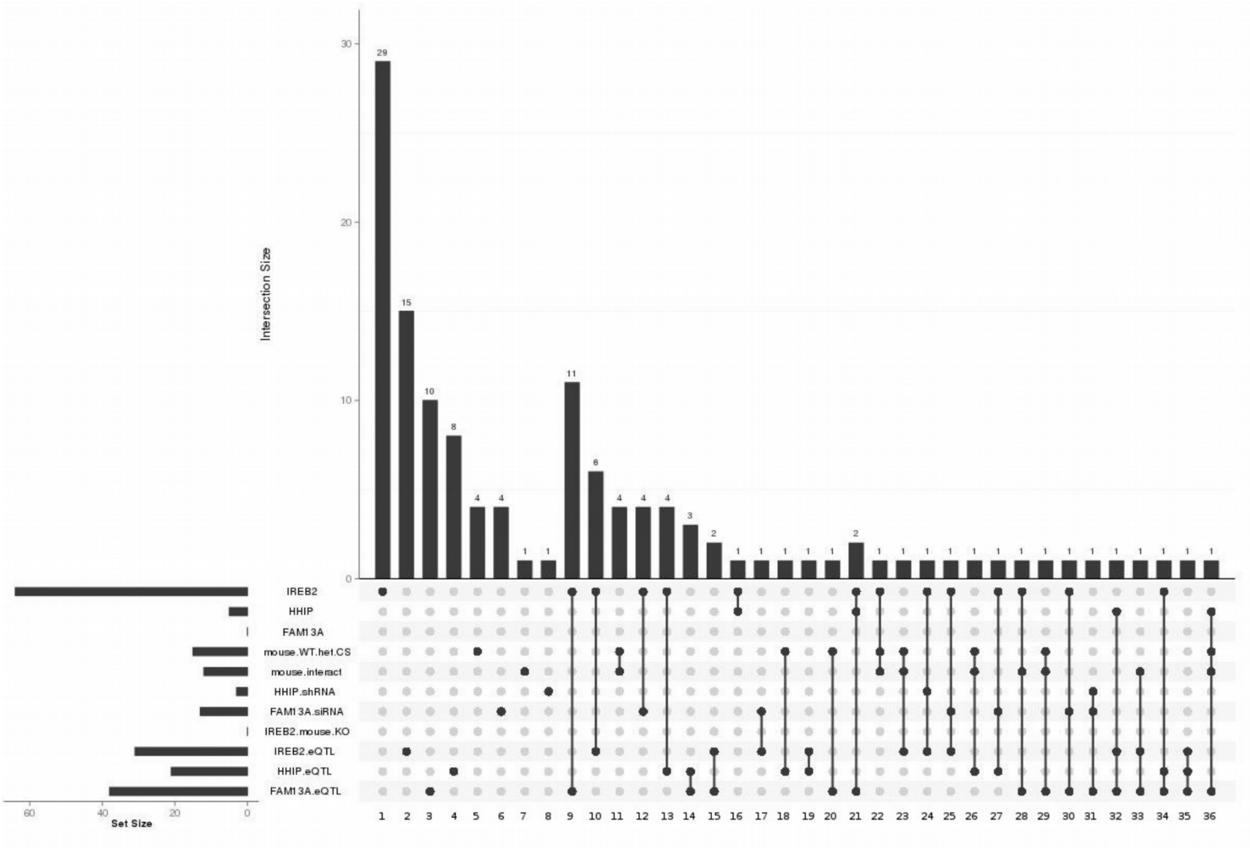

S2 Figure. Plot showing the intersection of overlap sets from the interactor enrichment tests for COPD differentially expressed genes. Each column in the plot provides Venn diagram type intersection content[41] (Supplemental Figure S3). Horizontal bars (Set Size) represent the size of overlap with the 204 COPD genes. Single solid dots represent genes that do not intersect with any other overlap set, and the solid dots connected by lines represent intersections. The vertical bars (Intersection Size) are proportional to the total number of genes for the solid dot(s) below them. For any given overlap set (any row), the total overlap count from Table 3 may be recapitulated by summing across all columns containing a solid dot. For example, the IREB2 enrichment overlap of 64 is found by summing columns 1, 9, 10, 12, 13, 16, 21, 22, 24, 25, 27, 28, 30, 34. The genes contained in each column count (value at top of bar) are listed in Table S8.

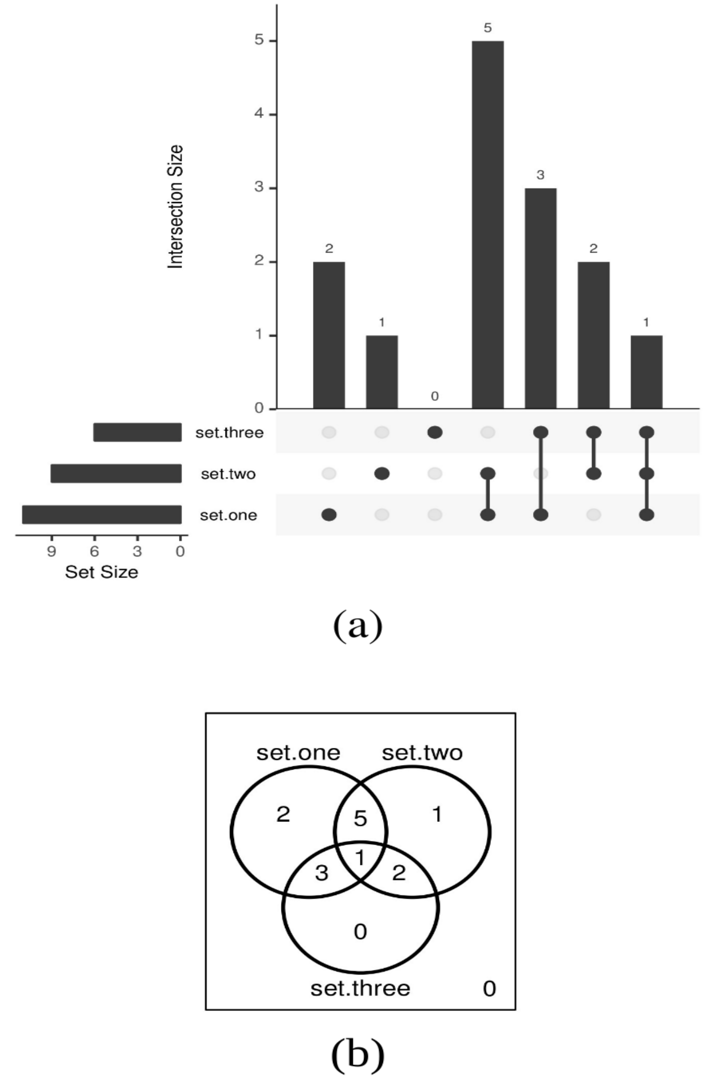

S3 Figure. A three-set example using hypothetical data to compare the presentations of intersections (a) UpSet plot and (b) equivalent Venn diagram. The complexity of presentation grows more quickly for a Venn diagram as the number of sets increases.
